# Development and Validation of a Competitive Risk Model in Elderly Patients With Chromophobe Cell Renal Carcinoma: A Population-Based Study

**DOI:** 10.3389/fpubh.2022.840525

**Published:** 2022-02-22

**Authors:** Jinkui Wang, Chenghao Zhanghuang, Xiaojun Tan, Tao Mi, Jiayan Liu, Liming Jin, Mujie Li, Zhaoxia Zhang, Dawei He

**Affiliations:** ^1^Department of Urology, Children's Hospital of Chongqing Medical University, Chongqing, China; ^2^Chongqing Key Laboratory of Children Urogenital Development and Tissue Engineering, Chongqing, China; ^3^Chongqing Key Laboratory of Pediatrics, Chongqing, China; ^4^Ministry of Education Key Laboratory of Child Development and Disorders, Chongqing, China; ^5^National Clinical Research Center for Child Health and Disorders, Chongqing, China; ^6^China International Science and Technology Cooperation Base of Child Development and Critical Disorders, Chongqing, China; ^7^Children's Hospital of Chongqing Medical University, Chongqing, China; ^8^Department of Urology, Kunming Children's Hospital, Kunming, China; ^9^Yunnan Provincial Key Research Laboratory of Pediatric Major Diseases, Kunming, China

**Keywords:** competitive risk model, nomogram, elderly, chromophobe cell renal carcinoma, SEER

## Abstract

**Background:**

Renal cell carcinoma (RCC) is the most common renal malignancy in adults, and chromophobe renal cell carcinoma (chRCC) is the third most common subtype of RCC. We aimed to construct a competitive risk model to predict cancer-specific survival (CSS) in elderly patients with chRCC.

**Methods:**

The clinicopathological information of the patients was downloaded from the SEER database, and the patients were randomly divided into the training and validation cohorts. Patients' risk factors for cancer-specific death (CSM) were analyzed using proportional subdistribution hazard (SH). We constructed a competitive risk model to predict the CSS of elderly chRCC patients. Consistency index (C-index), the area under receiver operating curve (AUC), and a calibration curve were used to validate the model's accuracy. Decision curve analysis (DCA) was used to test the clinical value of the model.

**Results:**

A total of 3,522 elderly patients with chRCC were included in the analysis. Patients were randomly assigned to either the training cohort (*N* = 2,474) or the validation cohort (*N* = 1,048). SH analysis found that age, race, T, N, and M stage, tumor size, and surgery were risk factors for CSM. We constructed a competitive risk model to predict patients' CSS. In the training set, the model predicted patients' 1-, 3-, and 5-year CSS with C-indices of 82.2, 80.8, and 78.2, respectively. The model predicted patient 1-, 3-, and 5-year CSS in the validation cohort with C-indices of 84.7, 83.4, and 76.9, respectively. The calibration curve showed that the model's predicted value is almost consistent with the observed value, which indicated that the model has good accuracy. The AUC of the training set and validation queue also suggested that the model has good discrimination. The clinical utility of the DCA model in predicting patients' CSS is higher than that of traditional TNM staging.

**Conclusions:**

We constructed a competitive risk model to predict CSS in elderly patients with chRCC. The model has good accuracy and reliability, which can help doctors and patients to make clinical decisions and follow-up strategies.

## Introduction

Renal cell carcinoma (RCC) is the most common renal malignancy in adults, with more than 400,000 cases diagnosed each year (1). According to its pathological classification, it is mainly divided into clear cell renal cell carcinoma (ccRCC), papillary renal cell carcinoma (pRCC) and chromophobe cell renal cell carcinoma (chRCC), etc. In 2016, The World Health Organization (WHO) defined chRCC as the third common subtype of RCC (2). ChRCC accounts for about 5–7% of RCC, with the same proportion in males and females, and the first diagnosis age of most cases is after 65 years old (3). Most patients are sporadic, and a few are hereditary, including BIRt-Hogg-Dube (BHD) syndrome (4) and tuberous sclerosis (TSC) (5). Comprehensive genomic analysis of chRCC showed a low mutation rate in its somatic cells, and the most common mutated genes were identified as TP53 and PTEN (6). ChRCC originates from the epithelial cells of the distal renal tubules, especially the α-intercalated cells of the renal collecting tubules. CcRCC derived from proximal renal tubular epithelial cells, which provided evidence for the heterogenesis of the two. ChRCC has a good prognosis, with a 5-year survival rate of 78–100% and a 10-year survival rate of 80–90% (7), which is significantly better than other RCCs, including ccRCC. So to this day, research on chRCC remains extremely rare. However, it is worth noting that 5% of chRCC patients are complicated with severe renal venous carcinoma thrombi (8), while nearly 10% of patients develop metastasis (9). The prognosis of these patients is similar to that of clear metastatic renal cell carcinoma, and the 5-year survival rate is only 14% (10). In addition, according to previous reports, factors determining the survival of chRCC patients also vary greatly (11, 12). Therefore, it is of great significance for the treatment and prognosis of chRCC patients to study influential prognostic factors, establish a high-precision prediction model and improve clinical treatment strategies for the particular group of the elderly, the central disease.

In recent years, with the broad application of the nomogram prediction model, traditional TNM staging has been gradually replaced. The prognosis of patients is also affected by many non-anatomic factors, such as age, gender, race, marital status, surgical methods, etc. (13). Nomogram is a data-based graphical computing tool that can estimate the risk of a disease based on staging systems such as the American Joint Commission on Cancer (AJCC) and other key risk factors related to prognosis (14). Today, many nomograms have been developed and applied to RCC, but mainly for patients with ccRCC and pRCC (15–18). However, there is still a lack of adequate and reliable nomograms to predict the prognosis of elderly patients with chRCC.

At present, artificial intelligence has been widely used in human health. Gadekallu et al. (19, 20) used deep learning to detect diabetic retinopathy. Kutia et al. (21) also discussed the great convenience that e-health systems bring to people. Kumar et al. (22) used neural networks to predict COVID-19. Iwendi et al. (23) use machine learning model for product recommendation. At present, there is no survival prediction model for chromophobe cell carcinoma in elderly patients. The existing prediction models are suitable for most of the population, but their accuracy is low.

Based on the above situation, we developed a competitive risk prediction model. We validated its accuracy in evaluating the cancer-specific survival rate (CSS) of elderly chRCC patients, providing a reference for clinical diagnosis and treatment.

## Methods

### Data Source and Data Extraction

We collected clinicopathological data from patients in the Surveillance, Epidemiology, and End Results (SEER) program of the National Cancer Institute. The SEER database is the national cancer database of the United States, covering ~28% of the US population and containing 18 cancer registries. Patient demographic information, clinicopathological information, and follow-up data can be obtained from the SEER database. Because the SEER database is a public database, we cannot identify patients' data, so our study does not require ethical approval and informed consent. Our methodology follows the rules of the SEER database.

**Figure 1:**
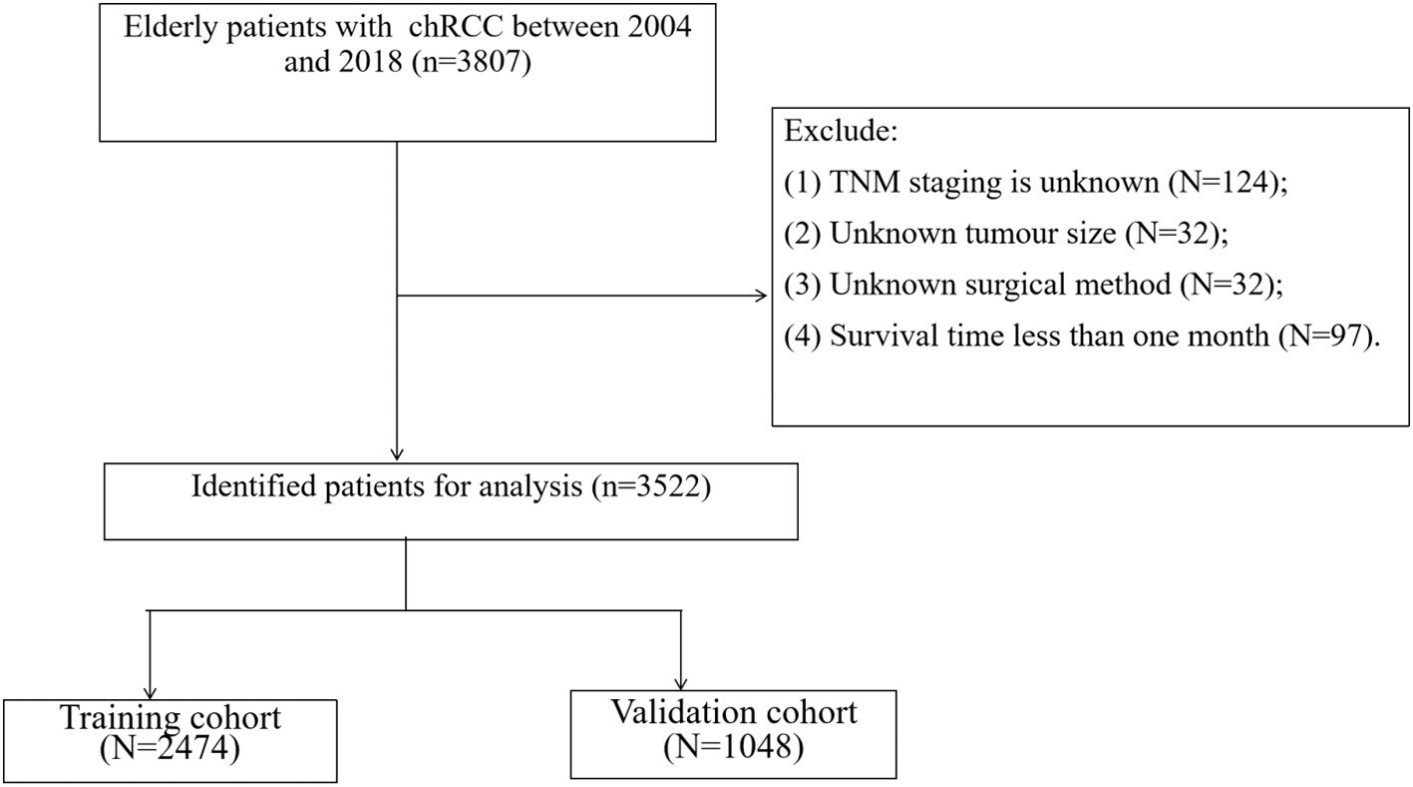
Flow chart of patient screening.

We collected clinicopathological information for all chRCC patients, including age, sex, year of diagnosis, race, marital status, tumor size, laterality, histological tumor grade, TNM stage, surgical method, radiotherapy, chemotherapy, survival status, and cause of death. Inclusion criteria: (1) Pathological diagnosis of chromophobe renal cell carcinoma (ICD-O-3 code: 8317); (2) Age ≥ 65; (3) Unilateral renal tumor. Exclusion criteria; (1) TNM staging is unknown; (2) Unknown tumor size; (3) Unknown surgical method; (4) Survival time <1 month. The screening process for all patients is shown in Figure 1.

Patients were divided into years of diagnosis from 2004 to 2010 and 2011 to 2018. Patients were categorized as white, black, and other (American Indian /AK Native, Asian/Pacific Islander). Histological grades of tumors include grade I (highly differentiated), grade II (moderately differentiated), grade III (poorly differentiated), and grade IV (undifferentiated). Patients' surgeries were classified into three categories, including local tumor excision (codes 10–27), partial nephrectomy (codes 30), and radical nephrectomy (codes 40–80).

### Construction and Validation of the Competitive Risk Model

At present, the main research methods of competitive risk include cause-specific risk model and cumulative risk model. The cumulative risk model takes into account other competing endpoint events when calculating a related endpoint event, which is closer to reality (24). All patients were randomly assigned to a training cohort (70%) or a validation cohort (30%). In the training cohort, the primary outcome was cancer-specific mortality (CSM), and the competing risk event was other causes of mortality (OCM). In the case of competition risk, the cumulative risk model is used to estimate the cumulative occurrence rate of interest events. Based on the proportional sub-distribution hazard model proposed by Fine and Gray, the influencing factors of cancer-specific death in patients were analyzed. At the same time, we used the risk factors of CSM to construct a competitive risk model to predict CSS. Consistency index (C-index), the area under the receiver operating curve (AUC), calibration curve were used to validate the accuracy and discrimination of the model.

### Clinical Utility

Decision curve analysis (DCA) was used to validate the clinical value of the model. We compared it with traditional TNM staging. All patients were divided into high-risk and low-risk groups based on competitive risk model scores. Log-rank test and Kaplan-Meier (K-M) curve were used to analyze survival differences between groups.

### Statistical Analysis

Measurement data (age and tumor size) were described by mean and standard deviation, and a non-parametric *U*-test analyzed differences. Count data were characterized by frequency (%), and the Chi-square test performed difference analysis or non-parametric *U*-test. We analyzed risk factors for CSM and death from other causes with proportional subdistribution hazard (SH). Log-rank test and Kaplan-Meier curve were used to analyze the difference in survival between groups. All statistical methods were analyzed by R software 4.1.0 and SPSS 26.0. A *P*-value < 0.05 was considered statistically significant.

## Results

### Clinical Features

A total of 3,522 elderly patients with chRCC were included in the analysis. Patients were randomly assigned to either the training cohort (*N* = 2,474) or the validation cohort (*N* = 1,048). The average age of the patients was 73.2 ± 6.11 years, and 2,907 (82.5%) were white, 2,035 (57.8%) were male, and 2,185 (62.0%) were married. There were 1,749 (49.7%) patients with tumors at the left side, and histological grades included 192 (5.45%) of grade I, 1,097 (31.1%) of grade II, 617 (17.5%) of Grade III, and 110 (3.12%) of grade IV. There were 2,974 (84.4%) patients in T1-T2, 3,470 (98.5%) patients in N0, and 3,452 (98.0%) patients in M0. The mean tumor size was 49.2 ± 35.0 mm. Local tumor excision was performed in 214 (6.08%) patients, partial nephrectomy was performed in 1,264 (35.9%) patients, and radical nephrectomy was performed in 1,903 (54.0%) patients. Fifty-five (1.56%) patients received chemotherapy and 23 (0.65%) received radiotherapy. The clinicopathological information of all patients is shown in Table 1. There was no significant difference between the training cohort and the validation cohort.

**Table 1:** Clinicopathological characteristics of elderly patients with chRCC.

	**ALL** ***N* = 3,522**	**Training cohort**	**Validation cohort**	***p***
		***N* = 2,474**	***N* = 1,048**	
Age	73.2 (6.11)	73.2 (6.13)	73.2 (6.07)	0.733
Race				0.879
White	2,907 (82.5%)	2,041 (82.5%)	866 (82.6%)	
Black	458 (13.0%)	320 (12.9%)	138 (13.2%)	
Other	157 (4.46%)	113 (4.57%)	44 (4.20%)	
Sex				0.333
Male	2,035 (57.8%)	1,416 (57.2%)	619 (59.1%)	
Female	1,487 (42.2%)	1,058 (42.8%)	429 (40.9%)	
Marital				0.899
No	1,337 (38.0%)	937 (37.9%)	400 (38.2%)	
Married	2,185 (62.0%)	1,537 (62.1%)	648 (61.8%)	
Year of diagnosis				0.824
2004–2010	1,302 (37.0%)	918 (37.1%)	384 (36.6%)	
2010–2018	2,220 (63.0%)	1,556 (62.9%)	664 (63.4%)	
Laterality				0.341
Left	1,749 (49.7%)	1,242 (50.2%)	507 (48.4%)	
Right	1,773 (50.3%)	1,232 (49.8%)	541 (51.6%)	
Grade				0.801
I	192 (5.45%)	133 (5.38%)	59 (5.63%)	
II	1,097 (31.1%)	786 (31.8%)	311 (29.7%)	
III	617 (17.5%)	431 (17.4%)	186 (17.7%)	
IV	110 (3.12%)	78 (3.15%)	32 (3.05%)	
Unknown	1,506 (42.8%)	1,046 (42.3%)	460 (43.9%)	
T				0.076
T1-T2	2,974 (84.4%)	2,107 (85.2%)	867 (82.7%)	
T3-T4	548 (15.6%)	367 (14.8%)	181 (17.3%)	
N				0.993
N0	3,470 (98.5%)	2,438 (98.5%)	1,032 (98.5%)	
N1	52 (1.48%)	36 (1.46%)	16 (1.53%)	
M				1.000
M0	3,452 (98.0%)	2,425 (98.0%)	1,027 (98.0%)	
M1	70 (1.99%)	49 (1.98%)	21 (2.00%)	
Tumor size	49.2 (35.0)	49.0 (36.1)	49.6 (32.4)	0.615
Surgery				0.642
No	141 (4.00%)	101 (4.08%)	40 (3.82%)	
Local tumor excision	214 (6.08%)	143 (5.78%)	71 (6.77%)	
Partial nephrectomy	1,264 (35.9%)	897 (36.3%)	367 (35.0%)	
Radical nephrectomy	1,903 (54.0%)	1,333 (53.9%)	570 (54.4%)	
Chemotherapy				0.968
No/unknown	3,467 (98.4%)	2,436 (98.5%)	1,031 (98.4%)	
Yes	55 (1.56%)	38 (1.54%)	17 (1.62%)	
Radiation				0.539
No/Unknown	3,499 (99.3%)	2,456 (99.3%)	1,043 (99.5%)	
Yes	23 (0.65%)	18 (0.73%)	5 (0.48%)	

### Construction of the Competing Risk Model

**Figure 2:**
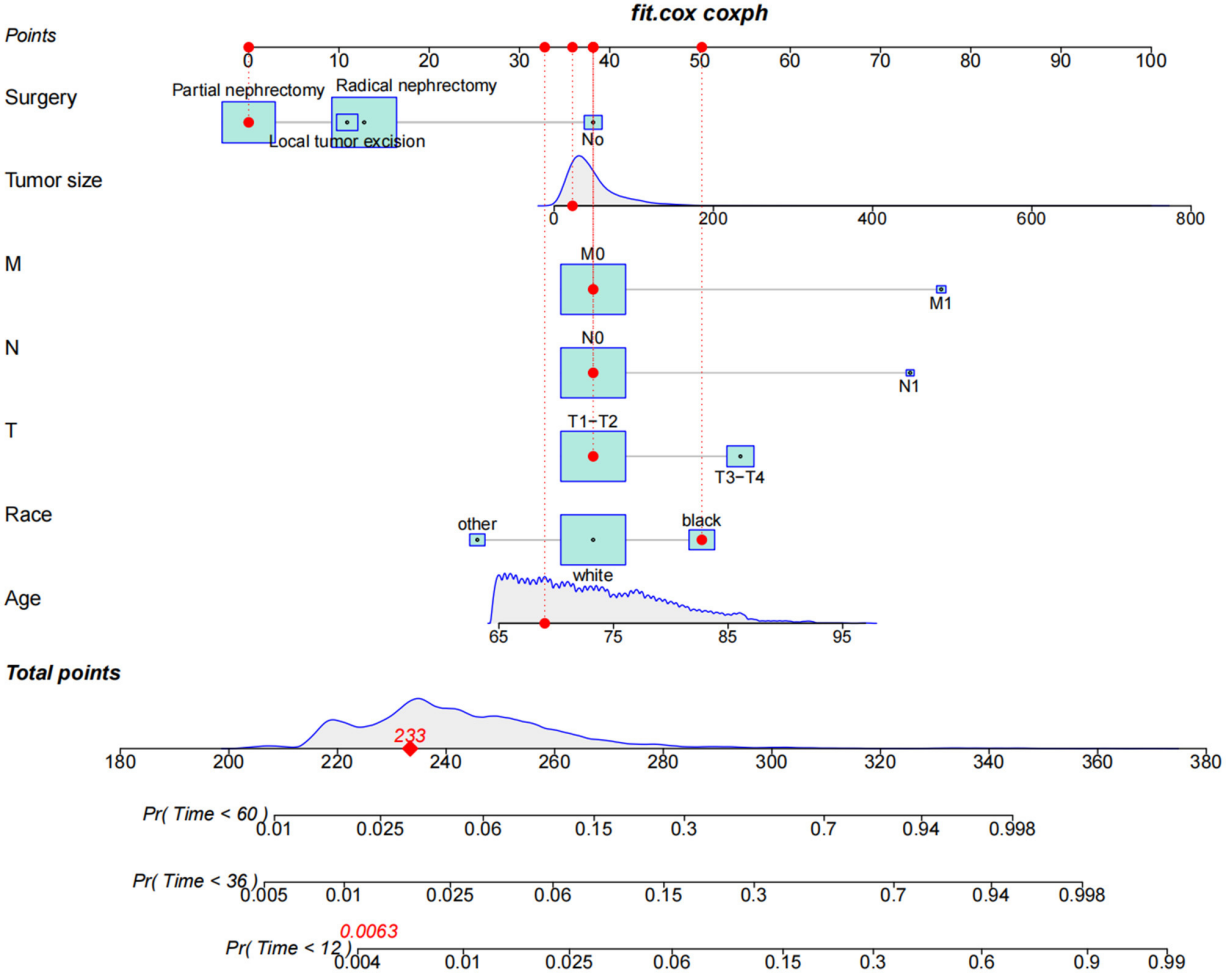
The competitive risk model nomogram of CSS in elderly patients with chRCC at 1-, 3-, and 5-year.

The training set analyzed the risk factors of CSM and OCM using SH and cumulative survival rate. The results showed that the risk factors of CSM in patients included age and race, T stage, N stage, M stage, tumor size, operation. Risk factors for OCM were age, sex, marital status, year of diagnosis, and tumor size (Table 2). We used patient risk factors for a cancer-specific end to construct a competitive risk model that predicted patient CSS (Figure 2). We developed a web page utility to calculate a patient's probability of CSS. Visit https://chenghao.shinyapps.io/DynNomapp/ to enter the site and a patient's clinicopathological features to obtain a CSS rate.

**Table 2:** Multivariate cox regression models predict cancer-specific mortality and other causes mortality in elderly patients with chRCC.

	**CSM**			**OCM**		
	**HR**	**95%CI**	***P***	**HR**	**95%CI**	***P***
Age	1.056	1.03–1.08	<0.001	1.076	1.06–1.09	<0.001
**Race**						
White						
Black	1.761	1.18–2.63	0.0055	1.238	0.95–1.62	0.12
Other	0.423	0.18–1	0.049	0.677	0.38–1.21	0.19
**Sex**						
Male						
Female	1.283	0.94–1.76	0.12	0.687	0.56–0.84	<0.001
**Marital**						
No						
Married	1.106	0.8–1.53	0.54	0.788	0.64–0.97	0.022
**Year of diagnosis**						
2004–2010						
2010–2018	0.772	0.56–1.06	0.11	0.786	0.63–0.97	0.028
**Laterality**						
Left						
Right	0.993	0.74–1.33	0.96	0.999	0.83–1.19	0.99
**Grade**						
I						
II	0.712	0.37–1.37	0.31	0.860	0.6–1.24	0.42
III	0.906	0.46–1.77	0.77	0.834	0.56–1.23	0.36
IV	1.850	0.85–4.02	0.12	0.586	0.3–1.14	0.12
Unknown	0.730	0.38–1.41	0.35	0.925	0.64–1.34	0.68
**T**						
T1-T2						
T3-T4	1.965	1.38–2.8	<0.001	0.975	0.75–1.28	0.85
**N**						
N0						
N1	4.552	2.38–8.71	<0.001	0.224	0.05–1.06	0.059
**M**						
M0						
M1	3.100	1.47–6.54	0.0029	0.522	0.12–2.22	0.38
Tumor size	1.004	1–1.01	<0.001	1.002	1–1	0.025
**Surgery**						
No						
Local tumor excision	0.346	0.15–0.82	0.017	0.920	0.47–1.79	0.81
Partial nephrectomy	0.197	0.11–0.37	<0.001	0.862	0.48–1.55	0.62
Radical nephrectomy	0.352	0.21–0.59	<0.001	0.855	0.48–1.52	0.59

### Validation of the Competitive Risk Model

**Figure 3:**
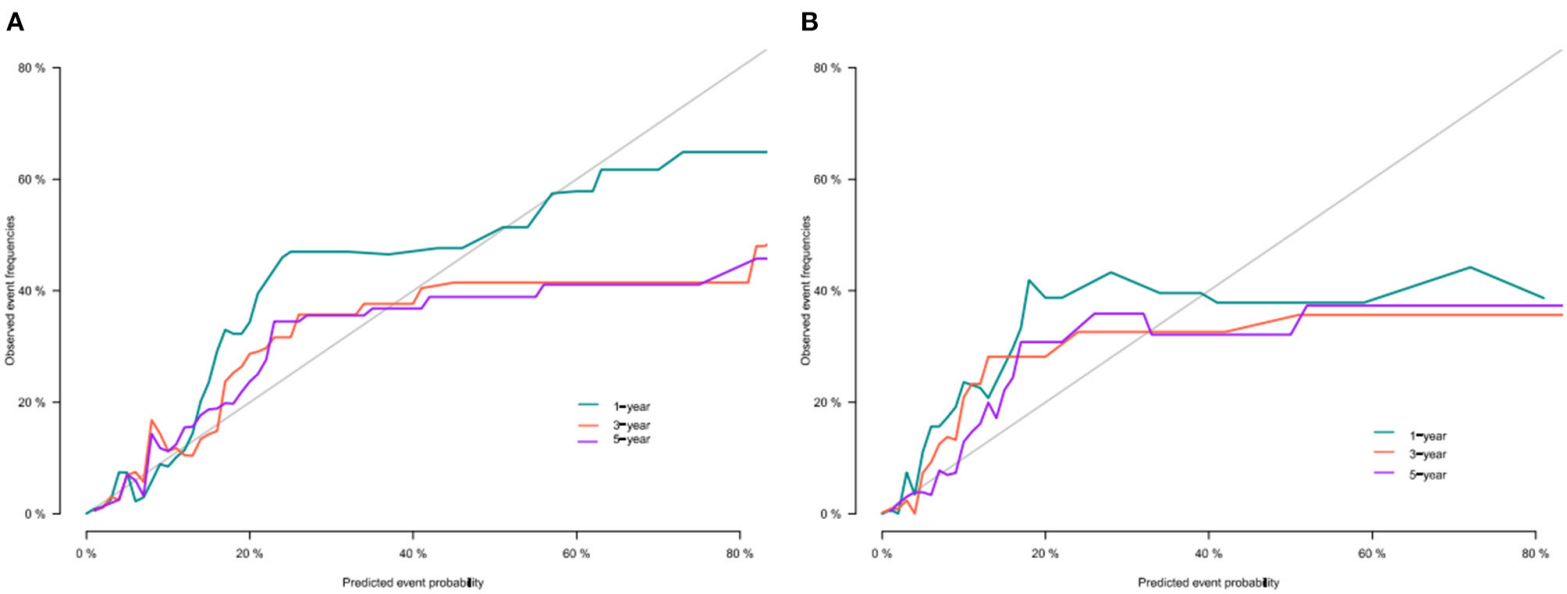
Calibration curve of the nomogram in training cohort **(A)** and validation cohort **(B)**.

**Figure 4:**
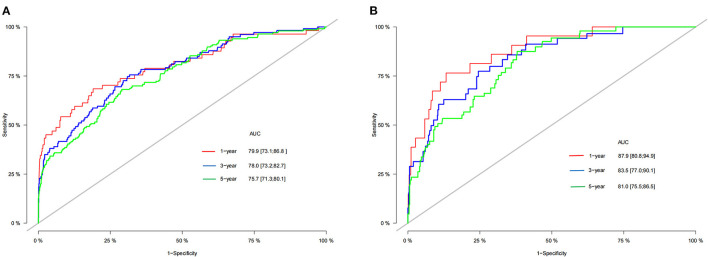
AUC for predicting 1-, 3-, and 5-year CSS in training cohort **(A)** and validation cohort **(B)**.

We use the C-index to validate the model's training and validation set accuracy. In the training set, the model predicted patients' 1-, 3-, and 5-year CSS with C-indices of 82.2, 80.8, and 78.2, respectively. The model predicted patient 1-, 3-, and 5-year CSS in the validation cohort with C-indices of 84.7, 83.4, and 76.9, respectively. The results show that the model has good discrimination. In addition, the model's calibration curve showed that the model's predicted value is almost consistent with the actual observed value, indicating that the model has good accuracy (Figure 3). The AUC of the training set and validation cohort also suggested that the model has good discrimination (Figure 4).

### Clinical Application of the Competitive Risk Model

**Figure 5:**
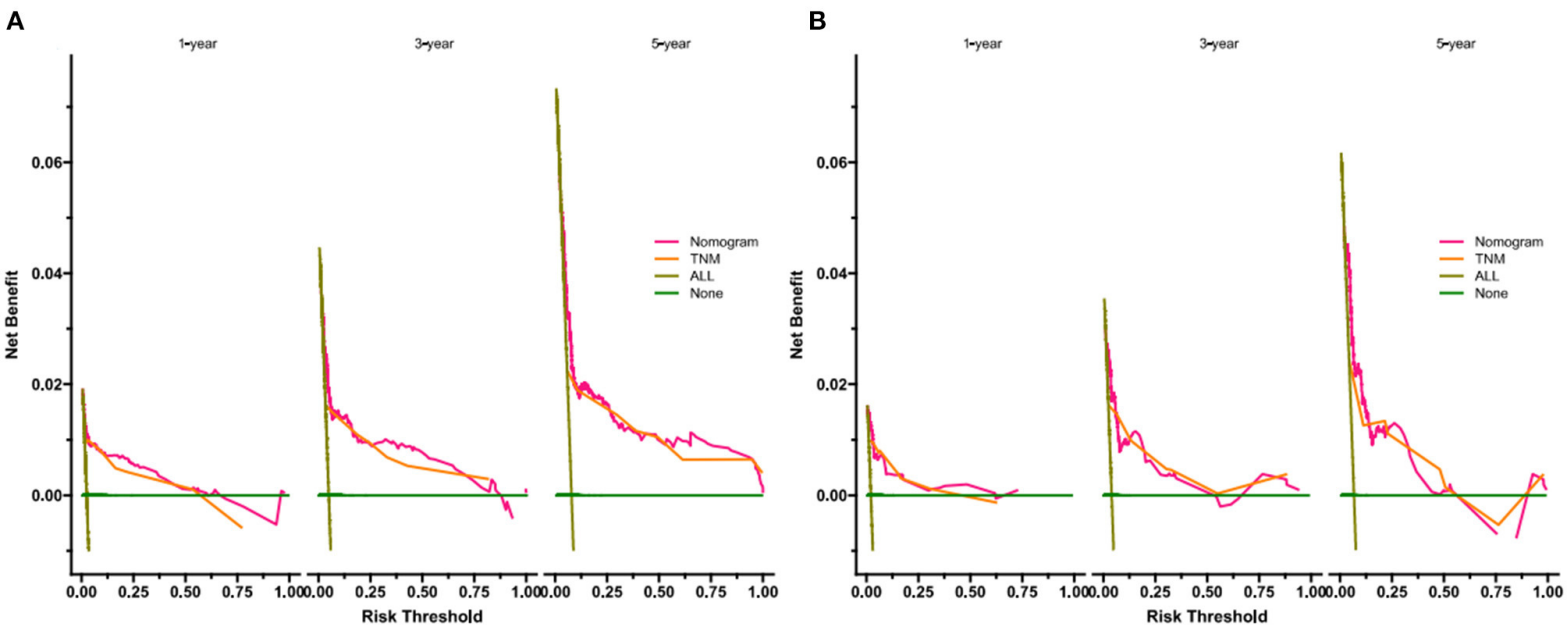
DCA of the nomogram in training cohort **(A)** and validation cohort **(B)**. The Y-axis represents a net benefit and the X-axis represents threshold probability. The green line means no patients died and the dark green line means all patients died. When the threshold probability is between 20 and 100%, the net benefit of the model exceeds all deaths or none.

**Figure 6:**
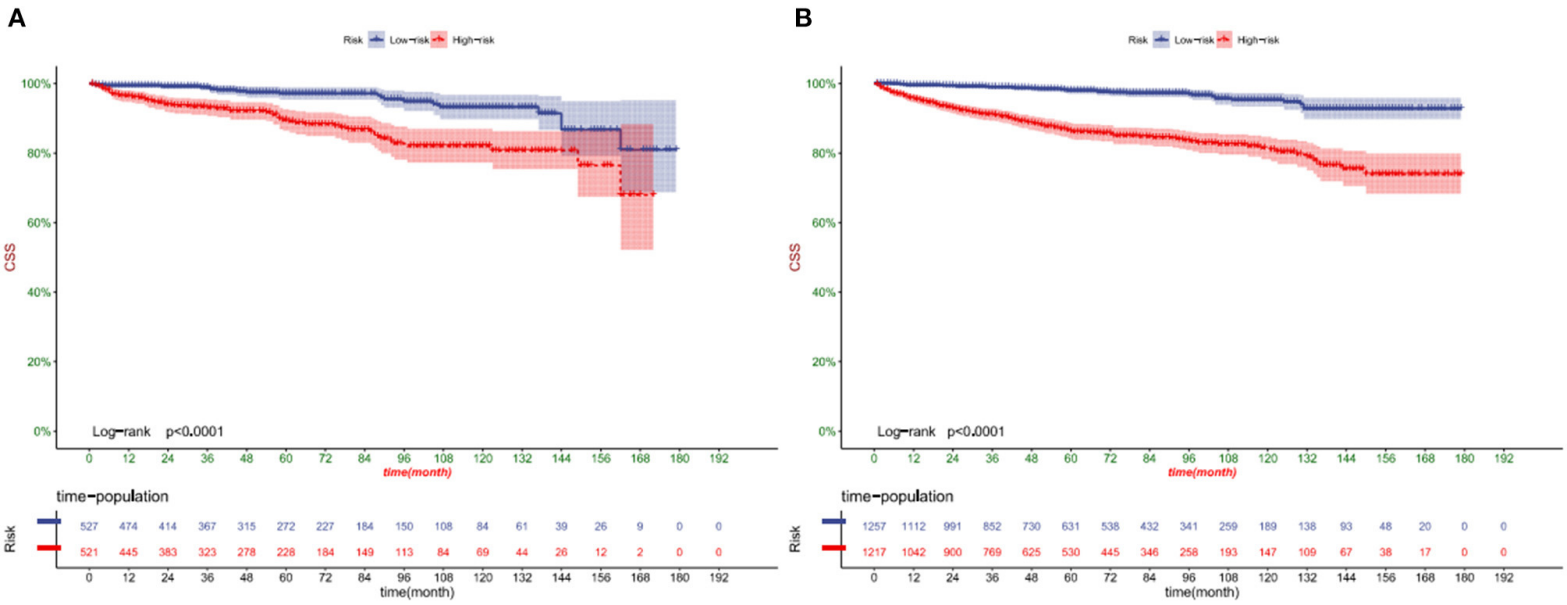
Kaplan-Meier curves of patients in the low-risk and high-risk groups in training cohort **(A)** and validation cohort **(B)**.

**Figure 7:**
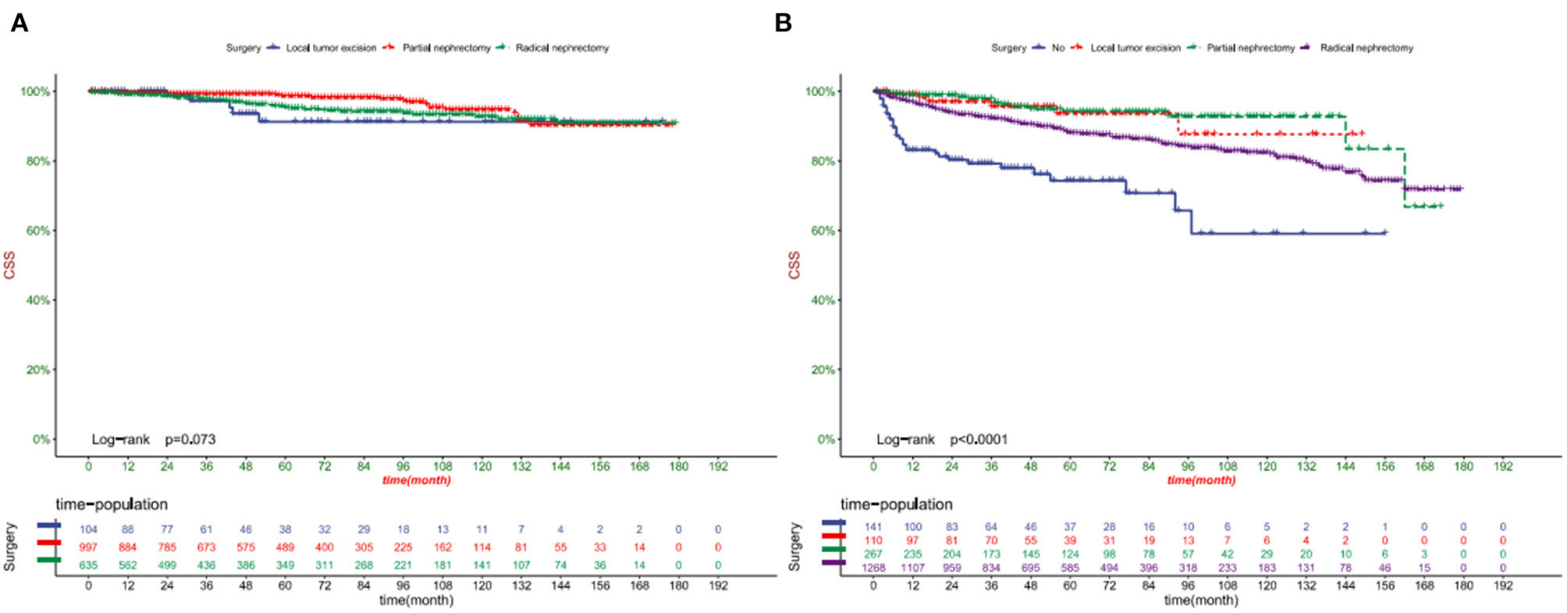
Kaplan-Meier curves of patients with different surgical procedures in the low-risk group **(A)** and high-risk group **(B)**.

DCA showed that in both the training and validation set, the clinical utility of the model in predicting patients' 1-, 3-, and 5-year CSS was higher than that of traditional TNM staging (Figure 5). We classified patients into the high-risk group based on their competitive risk model score (total > 54.1) and low-risk group (total score ≤ 54.1). K-M curve showed that the 1-, 3- and 5-year survival rates of patients in the high-risk group were 96.1, 92.3, and 88.2%, respectively, while the 1-, 3-, and 5-year survival rates of patients in the low-risk group were 99.1, 97.3, and 95.0%, respectively. In the training and validation sets, survival was significantly higher in the low-risk group than in the high-risk group (Figure 6). In addition, we analyzed surgical procedures in the high-risk and low-risk groups. In the low-risk group, all patients underwent surgery, and there were no significant differences in CSS between the types of surgery (Figure 7A). Most patients chose radical nephrectomy in the high-risk group, and patients with a partial nephrectomy and local tumor excision had significantly higher survival rates (Figure 7B).

## Discussion

chRCC is a new type of renal cell carcinoma (RCC) discovered by Thoenes et al. (25). The etiology of ChRCC is not clear yet, and some studies have claimed that it is closely related to chromosome variation. All chromosome loss is common in chRCCs, especially chromatids 1, 2, 6, 10, 13, and 17 (26). The inactivation of the TSC2 gene and the activation of MTOR is the most critical molecular changes (6). Under the pathological microscope, chRCC is characterized by large polygonal cells, pale cytoplasm, protruding cell membrane accompanied by raisin-like shrinkage (27). Ultrastructural analysis showed many abnormal mitochondria in the chRCC cytoplasm, with different sizes and shapes, accompanied by external swelling (28). In immunohistochemistry, CK7 is an important marker, expressed in more than 75% of chRCC, which can be preliminarily diagnosed by combining the diffuse positive expression of CD117 and KSP-cadherin (29). However, it still needs to be differentiated from eosinophilic carcinoma. ChRCC has a clear boundary and no capsule, only occurs in the kidney, and the section is gray or light brown. Eosinophilic carcinoma can occur in any part of the body, and the cut surface is peach-red (30, 31). ChRCC lacks typical clinical manifestations. A characteristic triad of low back pain, hematuria and abdominal mass rarely occurs in the early stage of ChRCC and is considered a marker of disease progression (32).

At present, the nomogram of renal cell carcinoma has been widely established. For example, Wang et al. (15) established a nomogram to predict the medium- and long-term prognosis of patients with papillary cell carcinoma. Peng et al. (16) established a nomogram to predict the prognosis of patients with clear cell carcinoma and renal cell carcinoma. Yan et al. (17) established a nomogram to predict cancer-specific survival in patients with papillary renal cell carcinoma. Chen et al. established a nomogram to predict the prognosis of chromophobe RCC patients, but did not involve competitive risk. Although these nomgorams can be used to predict survival in patients with renal cell carcinoma, they are not accurate enough. The competitive risk of survival in elderly patients with chRCC requires a more accurate prediction model.

The arrival of artificial intelligence makes big data medical care a reality. Maddikunta et al. (33) explore the potential application of artificial intelligence in industry 5.0. Healthcare 5.0 aims to use medical big data combined with artificial intelligence to assist human health. This study is to use the big data of cancer patients to establish a prediction model to predict the survival of patients.

Age is a critical factor in developing all cancers, and as we age, the risk of genetic mutations that could trigger cancer increases. Somatic mutations are generally considered the first step in the occurrence of cancer. They are associated with aging and highly reproducible DNA methylation changes, which help explain the higher prevalence of malignant tumors in the elderly (34). There is no consensus on defining the age of elderly patients, but more than 60% of initial cancer diagnoses and more than 70% of cancer deaths occur in patients over 65 years old (35). To improve the accuracy and representativeness of the prediction model, chRCC patients over 65 years old were included in this study. Escudier et al. (36) found that under the condition of the same stage and grade, there was a massive difference in the survival rate of patients and believed that clinical factors such as age were more critical than pathological and metastatic factors. Dias-Santos et al. (37) also showed that age is closely related to the survival rate of various cancers.

Surgery is a crucial measure for the treatment of chRCC, and tumor size based on clinical T-stage directly affects the choice of surgery and surgical method. Currently, the primary surgical procedures for chRCC include radical nephrectomy, partial nephrectomy, and local tumor excision represented by thermal ablation (TA), radiofrequency ablation (RFA), and cryoablation (CA). Current guidelines indicate that PN is still the standard recommended procedure for stage cT1a tumors, while there is controversy for stage cT1b, and RN is recommended for stage cT2 (38). However, with the increasing reports of chronic kidney disease (CKD) induced by RN and PN surgery, more and more attention has been paid to the requirement of nephron preservation, and tumor resection represented by TA is recommended to be used in cT1a stage RCC (39). TA is theoretically feasible for tumors larger than 4 cm, but it requires multiple puncture operations at various sites, and the probability of bleeding and other complications is significantly increased (40). At the same time, based on the solid support of extensive sample prospective studies, active surveillance (AS) is recommended for renal tumor patients with cT1 stage (41), with metastasis rates of 0–6% and CSS of 0–18% (42). It is worth noting that Huang et al. (43) found that in elderly chRCC patients over 65 years old, the mortality rate of the RN group was significantly higher than the PN group, which was presumed to be due to the risk of surgery and anesthesia caused by cardiovascular and cerebrovascular diseases and chronic kidney disease. Thompson et al. (44) also found a significant positive correlation between RN and mortality; therefore, AS has particular advantages in elderly patients.

In addition, the mean tumor size of ChRCC was 6.0 cm, which was significantly more significant than the other subtypes of RCC. However, the degree of malignancy was not higher than that of other RCCS, indicating no correlation between tumor growth and malignancy among different RCC subtypes (45). However, for the chRCC subtype, there was a linear relationship between tumor size and recurrence (46), which was consistent with this study. This study showed that the larger the tumor, the lower the patient's survival rate and could affect death from other causes.

This study is the first to construct a competitive risk model to predict cancer-specific survival in elderly patients with chromophobe cell carcinoma. This model has good accuracy and reliability through internal verification. However, there are still some shortcomings in our research. First of all, this study is a retrospective study, so it is challenging to avoid selection differences, which may cause a particular bias. Secondly, we did not include some key variables, such as BMI, smoking, drinking, hypertension, etc., which could improve the model's accuracy. Finally, our model has only undergone internal validation, and further external proof and future clinical application are necessary. Next, we will conduct the next prospective study to validate our prediction model.

## Conclusion

We identified age, race, TNM stage, tumor size, and surgery as risk factors based on competitive risk in elderly patients with chRCC. We constructed a competitive risk model to predict CSS in elderly patients with chRCC. The model has good accuracy and reliability, which can help doctors and patients to make clinical decisions and follow-up strategies.

## Data Availability Statement

Publicly available datasets were analyzed in this study. This data can be found here: https://seer.cancer.gov/.

## Ethics Statement

The data of this study is obtained from the SEER database. The patients' data is public and anonymous, so this study does not require ethical approval and informed consent.

## Author Contributions

JW and CZ designed the study. CZ, JW, JL, ML, LJ, and XT collected and analyzed the data. JW drafted the initial manuscript. CZ, TM, JL, ZZ, and DH revised the article critically. CZ, JL, DH, ML, and XT reviewed and edited the article. All authors approved the final manuscript.

## Funding

This work was supported by Special Key Project of Chongqing Technology Innovation and Application Development (No. Cstc2019jscx-tjsbX0003).

## Conflict of Interest

The authors declare that the research was conducted in the absence of any commercial or financial relationships that could be construed as a potential conflict of interest.

## Publisher's Note

All claims expressed in this article are solely those of the authors and do not necessarily represent those of their affiliated organizations, or those of the publisher, the editors and the reviewers. Any product that may be evaluated in this article, or claim that may be made by its manufacturer, is not guaranteed or endorsed by the publisher.
